# Novel methods included in SpolLineages tool for fast and precise prediction of *Mycobacterium tuberculosis* complex spoligotype families

**DOI:** 10.1093/database/baaa108

**Published:** 2020-12-15

**Authors:** David Couvin, Wilfried Segretier, Erick Stattner, Nalin Rastogi

**Affiliations:** WHO Supranational TB Reference Laboratory, Tuberculosis and Mycobacteria Unit, Institut Pasteur de la Guadeloupe, F-97183, Abymes, Guadeloupe, France; Laboratoire de Mathématiques Informatique et Applications (LAMIA), Université des Antilles, F-97154, Pointe-à-Pitre, Guadeloupe, France; Laboratoire de Mathématiques Informatique et Applications (LAMIA), Université des Antilles, F-97154, Pointe-à-Pitre, Guadeloupe, France; WHO Supranational TB Reference Laboratory, Tuberculosis and Mycobacteria Unit, Institut Pasteur de la Guadeloupe, F-97183, Abymes, Guadeloupe, France

## Abstract

Bioinformatic tools are currently being developed to better understand the *Mycobacterium tuberculosis* complex (MTBC). Several approaches already exist for the identification of MTBC lineages using classical genotyping methods such as mycobacterial interspersed repetitive units—variable number of tandem DNA repeats and spoligotyping-based families. In the recently released SITVIT2 proprietary database of the Institut Pasteur de la Guadeloupe, a large number of spoligotype families were assigned by either manual curation/expertise or using an in-house algorithm. In this study, we present two complementary data-driven approaches allowing fast and precise family prediction from spoligotyping patterns. The first one is based on data transformation and the use of decision tree classifiers. In contrast, the second one searches for a set of simple rules using binary masks through a specifically designed evolutionary algorithm. The comparison with the three main approaches in the field highlighted the good performances of our contributions and the significant runtime gain. Finally, we propose the ‘SpolLineages’ software tool (https://github.com/dcouvin/SpolLineages), which implements these approaches for MTBC spoligotype families’ identification.

## Introduction

Tuberculosis (TB) is an infectious disease caused by bacteria belonging to the *Mycobacterium tuberculosis* complex (MTBC), with a broad host range. MTBC includes a group of closely related species: *Mycobacterium tuberculosis sensu stricto, Mycobacterium africanum, Mycobacterium bovis, Mycobacterium caprae, Mycobacterium pinnipedii, Mycobacterium suricattae, Mycobacterium orygis, Mycobacterium microti, Mycobacterium mungi* and probably other ecotypes yet to be determined. Phylogenomic analysis of this group of organisms based on next-generation sequencing, digital DNA–DNA hybridization and average nucleotide identity showed that they might be considered as heterotypic synonyms of *M. tuberculosis* (1). TB is a global health problem that has killed 1.5 million people in 2018 according to the World Health Organization (2). Several genotyping methods are used to identify the MTBC isolates. Mycobacterial interspersed repetitive units—variable number of tandem DNA repeats (MIRU-VNTRs) and spoligotyping are two methods used mainly by researchers studying this pathogen (3, 4). Spoligotyping method uses the genetic diversity in the ‘clustered regularly interspersed short palindromic repeats’ (CRISPR) locus, which is also known as the direct repeat locus. More recent approaches are based on the Whole Genome Sequencing (WGS) to better decipher and classify the MTBC isolates. Seven major TB lineages have been identified: Lineage 1 (Indo-Oceanic), Lineage 2 (East-Asian), Lineage 3 [East-African-Indian (EAI)], Lineage 4 (Euro-American), Lineage 5 (West-Africa 1), Lineage 6 (West-Africa 2) and Lineage 7 (Ethiopian or Aethiops vetus lineage). These lineages are known to cause TB in humans throughout the world, and some of them (such as Lineage 3) are relatively specific to certain regions, whereas others (such as Lineage 4) are more globally distributed (5). In the SITVIT2 (6) proprietary database of the Institut Pasteur de la Guadeloupe (http://www.pasteur-guadeloupe.fr:8081/SITVIT2/), which is an update of previously released SpolDB/SITVIT databases (7, 8), Lineage 1 is known as EAI; Lineage 2 is known as Beijing; Lineage 3 is known as Central Asian (CAS); Lineage 4 includes Cameroon, Haarlem (H), Latin-American-Mediterranean (LAM), NEW-1 (formerly named Ural-2), S, T, Turkey, Ural and X; Lineage 5 is known as AFRI 2 and AFRI 3; Lineage 6 is known as AFRI 1 and Lineage 7 is known as Ethiopian. Two newly discovered lineages (Lineage 8 and Lineage 9) seemingly restricted to Africa were recently described (9, 10).

The design of prediction techniques for MTBC lineages is a critical task in TB molecular epidemiology because each lineage has its specificity and peculiarity regarding public health aspects such as virulence or antibiotic drug resistance. Therefore, knowledge concerning the lineages involved in a given outbreak may bring potential clues in TB diagnostics (by providing a better understanding of the disease) and in the development of improved treatments. Easily interpretable rules or methods allowing to differentiate lineages are constantly needed for a better understanding of TB epidemiological diversity in the given regions of the world. Data-driven methods could facilitate the accurate and rapid prediction of lineages based on an updated nomenclature. The automation of lineages prediction is also helpful for the curation/improvement of existing or novel biological databases.

In this paper, we focus on the classification of MTBC genotypic lineages. In particular, we propose two approaches that aim to extract interpretable models: (i) the decision tree (DT) with data transformation and (ii) the search for binary mask combinations with an evolutionary algorithm (EA). Our objective was to evaluate the performances of our two approaches in the prediction task of MTBC lineages. For this purpose, the performances of our methodology were compared to the three main classification approaches that stand as references in the field. The experiments conducted have thus allowed highlighting the good performances of our approaches, which are of great significance for the current database development and improvement.

## Related works

Knowledge Discovery from Databases (KDD) is the ‘non-trivial process of identifying valid, novel, potentially useful, and ultimately understandable patterns in data’ (11). Over the last 30 years, advanced prediction methods and tools borrowed to the KDD field have contributed to defining new kinds of prediction models, namely data-driven models. They consist of searching for correlations between predictive historical variables and output variables. When the output variables are discrete, the problems considered are referred to as ‘classification problems’. Among the current techniques used to tackle these problems, artificial neural networks (ANNs), DTs, instance-based learning or correlation analysis are the most common. They have proved to perform quite accurately in comparison to knowledge-driven solutions with the advantage of requiring less knowledge for their implementation. However, one of the issues in data-driven approaches is the understandability and readability of models that end-users should trust. Indeed, a lot of techniques, including ANNs, can be seen as delivering black-boxes since they do not provide explanations of how they work. Decision-makers are more likely to trust models whose predictions are interpretable and understandable.

EAs (12, 13) are population-based metaheuristic optimization methods inspired by the main principles of the neo-Darwinian theory of evolution. They have been widely used in the KDD field (14), including the search of rule-based prediction models (15, 16). A population of solutions undergo evolution by applying genetic operators such as selection, replacement and mutation. These methods have proved to be efficient to find useful solutions for optimization and search problems with acceptable execution time comparatively to exhaustive techniques. In the following section, we present the main parts of the EA that we have designed to address our classification problem.

Several software tools allow to predict MTBC genotyping families from spoligotyping and/or MIRU-VNTR patterns; examples include the following online resources: MIRU-VNTRplus (17), StackTB (18), TB-Lineage and other TB-Insight’s tools (19) and TBminer (20); however, most of the existing tools are only available as online tools limiting the amount of data that can be analysed. Some available software tools are using more or less complex algorithmic approaches that are not easily understandable for neophytes (or persons with few or no computing skills). All the available tools are nevertheless useful and present specific functionalities and complementary prediction algorithms.

RuleTB is one of the approaches used in StackTB online tool, allowing to determine MTBC families from 24-loci MIRU-VNTR basing on a direct method that proposes a concise set of rules (18). StackTB also uses a machine learning approach that requires only a fraction of training data to determine lineages. RuleTB is used in SpolLineages to potentially predict MTBC families from MIRU-VNTR data.

TB-Lineage is an online tool using a rule-based system allowing to classify MTBC genotypes (spoligotyping and/or 24-loci MIRU-VNTR) into major phylogenetic lineages. The model was trained using labelled spoligotype data with spoligotypes belonging to East Asian, EAI, Euro-American, West African 1 and 2, *M. bovis* and Indo-Oceanic lineages. For the purpose of the Naive Bayes classifier, each spoligotype is represented as a binary 12-dimensional feature vector. Each dimension represents the presence/absence of a contiguous deletion. Presence of a deletion means no spacers are present in the subsequence, while absence means at least one spacer is present in the subsequence. The model takes into account the fact that the evolution of the direct repeat (or CRISPR) locus occurs via deletion of one or more contiguous spacers with some non-negligible probability, whereas insertion of repeats is highly unlikely (22). The features selected to represent a spoligotype were single deletions of spacers 3, 16, 8, 9 and 39 and contiguous deletions of spacers 1–34, 25–28, 29–32, 33–36, 39–43, 4–7 and 23–24 (19).

Borile_AP (23) is an affinity propagation algorithm embedded in TBminer (20), allowing to assign MTBC spoligotype families from spoligotyping data. Furthermore, TBminer also utilizes different machine learning algorithms to predict a consensual taxonomy from lineage assignations obtained using TB-Lineage, MIRU-VNTRplus and SITVITWEB. In the Borile affinity propagation taxonomy, distances to the 32 Borile references were computed based on shared blocks of absent spacers (23). Every isolate was assigned to the group of the most similar reference, and unassigned when equal distances were found with at least two references. Borile AP taxonomy also used three methods to compute distances: Domain Walls, Blocks and Deletions methods. These distance methods were used to compute the distance between each SpolDB4 spoligotype pattern and the references of the main MTBC families. The AP method is based on the choice of ‘exemplars’ as centres of the clusters, i.e. one representative data point for each cluster to which the other nodes rely upon. The choice of the exemplars is based on the minimization of the total ‘energy’ of the system, function of the total distance between data points and exemplars in a given clusters configuration. This method falls in the class of message-passing type algorithms, exploiting the belief propagation method (also known as cavity method) to minimize the energy function in an computationally efficient way [from the exponential time complexity of the naive methods to *O*(*N*^2^), where *N* is the total number of nodes to a cluster]. The starting point is thus a set of data points, representing the nodes of the network, and a similarity matrix *S* defining the similarities among all the nodes as deduced from the distance between all these nodes (20).

Researchers can also use the SITVIT2 database and its associated tool SpolSimilaritySearch (24) to query the database or search for similar spoligotype patterns from the database using regular expressions. The binary rules described in SITVIT2 (defined here as expert rules) were used with regular expressions taking into account 43 spoligotyping spacers, where the character ‘n’ (or number 1) represents a mandatory presence of spacer, the character ‘o’ (or number 0) represents an absence of spacer and the character ‘.’ represents either an absence or a presence of spacer. When a given spoligotyping pattern is classified ambiguously and corresponds to different families, the newly defined priority rules consist in first selecting the families that would be the most specific (basing on our past experience in spoligotyping patterns analysis). For example, a spoligotyping pattern that has been classified as belonging to both T1 (defined by the absolute presence of spacer rank number 31 and the absence of spacer rank numbers 33–36) and X1 (defined by the absolute presence of spacers 17 and 31, and by the absence of spacer 18 as well as spacers 33–36) sublineages would be reclassified as belonging to X1 sublineage since X1 prototype is more specific than T1 family according to the priority rules implemented in the SpolLineages program. Another example is the classification of patterns potentially belonging to both T and Turkey lineages, where the priority rules will allow the selection of Turkey if this ambiguity is noticed (see binary rules table on the dedicated ‘Help’ page of SpolLineages website for further details). Table 1 shows some examples of binary rules for MTBC spoligotype families (AFRI 2, Beijing, CAS1-Delhi, EAI1-SOM, Ethiopian, H1, LAM1, T1 and Turkey) used in the SITVIT expert classification.

**Table 1.** Examples of expert binary rules for some MTBC spoligotype families

Sublineages	43 spoligotyping spacers
AFRI 2	……nooooon……noooon……….nooon…
Beijing	oooooooooooooooooooooooooooooooooo………
CAS1-Delhi	..noooon………….noooooooooooon……..
EAI1-SOM	……………………….oooono. non..
Ethiopian	nnnooooooooooooooooooooon..oo……n…….
H1	……………………noooooonoooo…….
LAM1	..o……………..oooon…..n.oooo…….
T1	…………………………n.oooo…….
Turkey	……………….ooooonoon..n.oooo…….

Further details concerning spoligotyping based MTBC families/lineages could be found in previous works (6–8).

Interestingly, other software tools were developed, allowing to predict spoligotyping and MIRU-VNTR patterns from WGS data (SpolPred, SpoTyping, MIRU-profiler and MIRUReader) (25–28). Furthermore, the following programs are some examples of tools allowing to make lineage and/or antibiotic resistance prediction from WGS FASTQ (https://en.wikipedia.org/wiki/FASTQ) sequence reads: KvarQ, TB-Profiler, PhyResSE and TGS-TB (29–32). Another recent software named SNP-IT allows predicting MTBC lineage directly from DNA sequence using single-nucleotide polymorphisms (SNPs) (33).

## Methodology

Datasets were extracted from the SITVIT2 database. A subset of 7668 (4594 for the training set + 3074 for the testing set) unique profiles were randomly generated from strains belonging to 14 representative MTBC spoligotype families as defined in SITVIT2 (6). Predictions from NEW-1 (previously misnamed as Ural-2 in SITVIT2 database) were intentionally included in the Ural family for the analyses. Predictions from Borile AP and TB-Lineage were collected using TBminer (which provides a greater capacity of input file size). The current solution for classifying MTBC spoligotype families in SITVIT databases is based on spoligotyping binary rules. Spoligotyping-based binary rules exist since previous versions of SITVIT databases. These binary rules have been updated in the more recent versions of the database in the function of current and ongoing findings concerning the MTBC families. These binary rules were generally used to classify TB strains in the function of their spoligotyping patterns. Users can either (i) use these rules directly for manual annotation/curation of their data (using other metadata when necessary); (ii) query the SITVIT2 database to check if their data were already detected elsewhere or (iii) use other online tools such as StackTB, TBminer, TB-Lineage or MIRU-VNTRplus to predict lineages from their data. In the improved approach using expert binary rules, RuleTB (18) refined rules were used to predict lineages from 24-loci MIRU-VNTR data. Data on 24-loci MIRU-VNTR from our datasets were not sufficient to conduct a comparative study as was done for spoligotypes. This is why RuleTB was used as an intermediate method (more pending data are available on 24-loci MIRU-VNTR).

### Reducing spacers for DT-based modelling

One of the first approaches we propose to obtain a model that can be easily interpreted by humans is ‘DTs’-based modelling. Indeed, DTs have long been used in various predictive tasks and have the advantage of being readable and quick to execute (34).

**Figure 1.**
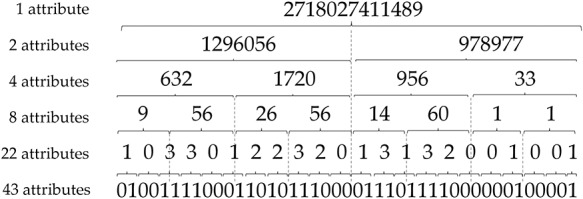
Spacer reduction strategy for predictive modelling with DTs.

In this work, we are interested in DTs for their ability to simply describe the underlying model as a set of rules. These rules are then synthesized in a graph structure, in which each leaf corresponds to a predicted class and each path to a leaf corresponds to a rule. As our goal is to provide rules that can be easily interpreted by a human, we have attempted to reduce the initial 43 spacers by performing dichotomous mergers as described in Figure 1.

More specifically, to reduce the number of attributes, we convert the binary number resulting from the concatenation of the spacers to an integer. By this way, the fusion of the initial 43 spacers allow to obtain six different datasets successively containing 43, 22, 8, 4, 2 and even only 1 attributes.

Thus, the objective of this attribute reduction step is twofold: (i) to evaluate whether the attribute reduction can maintain satisfactory predictive performance and (ii) to obtain DTs with simple predictive rules that involve the fewest attributes.

### Search for binary masks

In this section, we present the second original method that we developed to classify tuberculosis spoligotypes. As seen earlier, spoligotypes are represented by sets of 43 spacers materialized by binary digits. The main idea of this second approach is to use combinations of binary masks to distinguish spoligotype families. The search for such combinations of binary masks can be seen as a combinatorial optimization problem which search space *S* is the set of all possible combinations of *n* masks. We propose to use an EA to tackle this optimization problem and find the best combinations.

The classification of spoligotype families is a multiclass classification problem as there are 14 families to distinguish. However, as we will see in the rest of this section, our approach is binary by nature since we determine if a spoligotype belongs to a given family or not. In ‘EA model and parameters’ section, we will explain how individual binary classifiers are integrated into a single 14-class classifier through a ‘One-vs-All’ (OVA) strategy.

#### Individual representation

**Figure 2.**
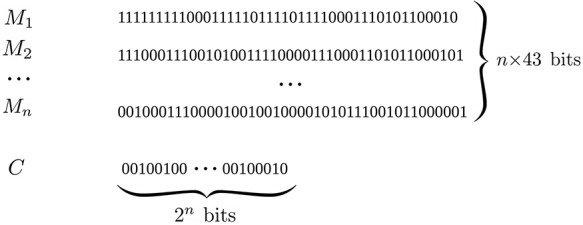
Binary representation for an EA solution.

A solution (individual) *s* represents a binary classifier taking into account (as input) spoligotype spacers binary strings *Sp* and making a decision as output: either a given family is detected (positive) or not (negative). The classifier consists of *n* 43 bits’ binary masks *M_i_* and a 2*^n^* bits binary string *C*. Figure 2 shows an example of a solution using this representation. In order to make a prediction, each mask *M_i_* is applied separately to *Sp* through a bitwise AND operator, resulting either in a null or a non-null binary value *R_i_*. Rules based on logical conjunctions of *R_i_* values are then derived from *C*. Indeed, as there are 2*^n^* ways to combine *n* binary values, each bit of *C* corresponds to an output class for a given rule. Table 2 gives some insights about the association between a set of rules and an individual. Each line corresponds to a rule. The first column shows the conjunction of *R_i_* values; the second column shows the bit of *C* associated with the rule and the last column shows the output class corresponding to that bit. When a conjunction is true, the classifier answers with the specified class. According to this individual representation, the size *S* of the search space is given by the following formula:

$$\begin{equation*}\left| s \right| = {2^{{2^n}}} \times \prod\limits_{i = 1}^n {{2^{43}}} \end{equation*}$$

**Table 2.** Set of rules corresponding to an EA solution with *n* = 2

Conjunctions (*R_i_* = *M_i_* AND *Sp*)	C bits	Output class
*R*_0_ = 0 ∧ *R*_1_ = 0	1	Beijing
*R*_0_ = 0 ∧ *R*_1_ > 0	0	Other
*R*_0_ > 0 ∧ *R*_1_ = 0	0	Other
*R*_0_ > 0 ∧ *R*_1_ > 0	1	Beijing

With *n *= 4, we obtain *|S| ≈ *3.92 *× *10^56^.

#### Genetic operators

Genetic operators are crucial in an evolutionary process as they guide the evolution of a randomly generated initial population toward the best reachable solutions. These operators can be classified in two categories. Problem-independent operators such as selection or replacement are generic operators that can be used in any EA regardless of the problem considered. They see individuals as a whole and use their fitness value to achieve their treatment. The choices that we made regarding these operators will be presented in ‘EA model and parameters’ section, where we show the parameters that we used and the results that we obtained. In contrast, problem-dependent operators such as crossover or mutation highly depend on the representation chosen as they need to create new individuals from the genotype of existing individuals. In this sense, they are often called variation operators. The crossover operator is the analogue of reproduction: it takes two individuals as input—the parents—and generates two new individuals—the offspring. The mutation operator is equivalent to the biological mutation: it creates a new individual by altering a small part (gene) of an existing individual to maintain the genetic diversity. As our individuals are made of binary strings, which have been widely used in EA, we can use classical binary variation operators with no constraints on the generated strings. We use a two-point crossover where two positions are selected randomly from the parents and the bits in between these two points are swapped to create the offspring. The *i^th^* mask of the first parent is crossed with the *i^th^* mask of the second parent. The same goes for the *C* value of each parent. The mutation operator randomly chooses one position of a binary string and flips its value. It is also applied to each mask and the C string of an individual.

#### Objective function

The role of the objective function, often called fitness function in the EA vocabulary, is to assign a quality value to an individual to implement the ‘survival of the fittest’ aspect of the evolutionary process. The choice of this function is extremely important as individuals with good values will be favoured during the process. In a classification problem, the most straightforward choice for this function is the precision of the classifier on the learning dataset, i.e. the percentage of its right answers, which gives an overall performance measure. However, as we use an OVA strategy, the relabelled datasets on which each binary classifier works are necessarily quite imbalanced, with the ‘family’ class being much less represented as the ‘Other’ class. In this case, the search for individuals with good precision values would lead to classifiers with better proper negative rates. It is the reason why we use the ‘Matthews Correlation Coefficient (MCC)’ (35) defined as follows:

$$\begin{equation*}MCC = {{TP \times TN - FP \times FN} \over {(TP + FP) \times (TP + FN) \times (TN + FP) \times (TN + FN)}}\end{equation*}$$

where TP is the quantity of true positive, TN is the quantity of true negative, FP is the quantity of false positive and FN is the quantity of false negative. It offers the advantage to be particularly well suited for imbalanced datasets.

### Comparison approach

Our objective in this paper is to evaluate the performances of our two approaches in the prediction of MTBC spoligotype families: (i) the DT with the reduction of spacers and (ii) the binary masks with the EA. To evaluate the efficiency of these approaches, we compare the performances with the three main classification approaches that are references in the field: ‘Expert rules’, ‘TB-Lineage’ and ‘Borile AP’, described in ‘Related works’ section.

**Figure 3.**
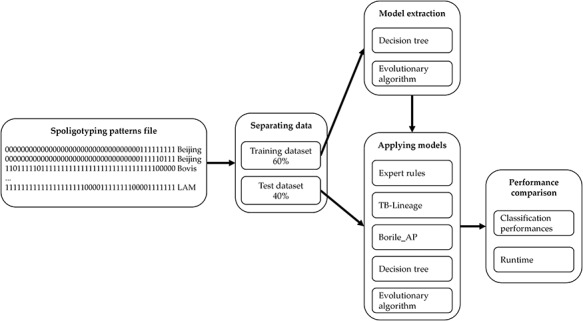
Methodology for comparing classification models.

Thus, the comparison process we adopt performs in five steps as described in Figure 3. We start from a spoligotype file that contains multiple samples of spacers for each MTBC spoligotype family. We then separate the dataset into two parts: 60% of the samples are used for generating a training set while the remaining 40% are used for testing. The training set is used for modelling with DT and EA as described previously. Finally, the models learned are applied to the testing set to compare the results with ‘Expert rules’, ‘TB-Lineage’ and ‘Borile AP’. The quality of the different models is compared by using several performance indicators such as the true positive (TP) rate, the false positive (FP) rate, the precision and the average error rate. We compare the performances on each of the 14 MTBC spoligotype families addressed and we also focus on the average of the results to study and compare the overall performances of the models. To go further, we compare the execution time to evaluate the ability of models to analyse large datasets. Finally, we show some examples of the rules used by our models to perform classification.

## Experimental results

### Impact of spacer reduction on DT performances

**Figure 4.**
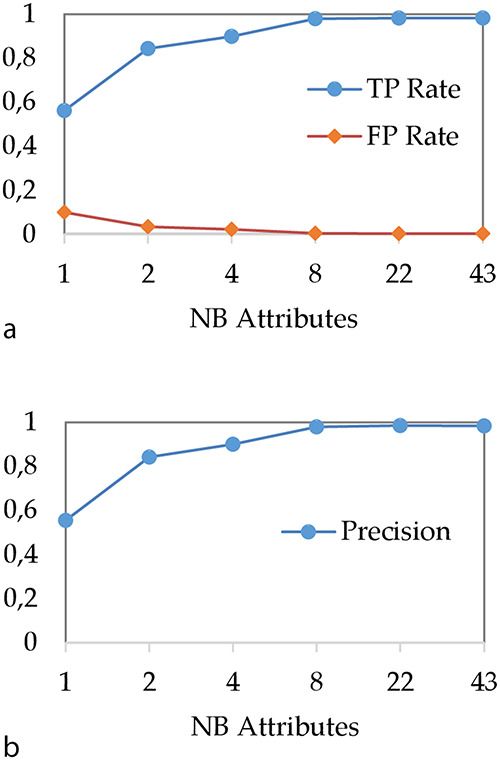
Impact of attribute reduction step on DT performances (a) TP and FP rates and (b) precision.

In a first step, we have studied the impact of the spacer reduction step on the DT performances. Our objective was to reduce the model creation phase, to simplify the underlying classification rules and to evaluate the impact of this procedure on performances. Figure 4 shows the evolution of (i) TP and FP rates and (ii) precision when the number of attributes used for spacer reduction is evolving. In our experiment, we use ‘C4.5’ algorithm (36) for modelling with a DT, with the minimum number of instances of object per leaf set to two and confidence factor set to 0.25.

As expected, we can observe that the performances of the DT increase with the number of attributes. In particular, when the number of attributes is very low, the DT does not perform well. For instance, when all 43 spacers are aggregated to a single integer attribute, TP rate is about 56% while precision is about 55%. These bad results can be explained by the fact that a too brutal spacer aggregation causes an important loss of information that does not allow any more to correctly classify MTBC spoligotype families. The most important result concerns the performance we obtain when 43 spacers are aggregated over eight attributes. Indeed, with eight attributes, the TP rate and precision are about 98%, which is equivalent to the results obtained when all 43 spacers are considered.

Thus, these results show that the DT can be used with a reduction of 43 spacers on only eight attributes without loss of performance. Therefore, in the rest of the article, we compare the DT with other models by using 8 and 43 attributes.

### EA model and parameters

We have conducted numerous experiments to find the right parameters for our EA. The first choice we had to make was about the number of masks *n*. A too small value would not have allowed finding optimal solutions, as the number of possible rules would have been too low. At the same time, a too high value would have significantly increased the size of the search space|*S*|, making it harder to reach optimal solutions in a reasonable time. As a fair trade-off, we have chosen the value *n *= 4. One common problem in EA is ‘premature convergence’ (37), i.e. the fact that the population converges too early resulting in suboptimal solutions. Indeed, as the population begins to evolve through the use of genetic operators, the building blocks of the current best individuals spread relatively quickly to the rest of the population, leading to a reduction of the genetic diversity. However, the preservation of a certain amount of diversity is crucial when it comes to finding new best individuals. It is typically the role of the mutation operator, as explained in ‘Search for binary masks’ section, whose application rate may vary. To go further, we also modified our crossover operator in a way that when two identical parents are selected to mate with each other (which would generate two identical offspring), one of the offspring is replaced by a new randomly generated individual. It is known as a ‘headless chicken crossover’ (38) and can be seen as a form of macro-mutation. Another way to combat premature convergence is the use of low selection pressure, allowing non-optimal solutions to survive and transmit their building blocks throughout the generations. It is why we used a binary tournament selection operator, consisting of choosing the best solutions among sets of two individuals with a probability *p* for reproduction. Finally, to further improve our results, we decided to use an island model parallel EA (39). The main idea of this model is to evolve simultaneously several isolated populations. From every *k* generations, the best individual of each island is sent to other islands to enhance the quality of their respective populations. In addition to allowing better preservation of genetic diversity, this model offers two interesting advantages:

It allows to take advantage of the parallel architecture found in most recent microprocessors, leading to either significant runtime reduction or the increase in the total number of considered individuals.
It allows using simultaneously different parameters (crossover rate, mutation rate, tournament selection probability, etc.) for the evolution of each island; indeed there are no predefined correct values for these parameters that would suit every optimization problem.

Table 3 summarizes the choices that we made for our EA parameters. *n* is the number of masks of a binary classifier, *islands* is the number of islands used in our parallel EA, *gens* is the number of generation during which the process takes place and *migration* is the number of migrations, i.e. exchange of best individuals, between the islands during the evolution (a migration occurs in every *gens/migration* generation), *pcross* is the crossover rate, *pmut* is the mutation rate and *p* is the binary tournament selection probability. Notice that these three last elements are represented by intervals: a value comprised in these intervals has been affected by each island.

**Table 3.** EA parameters

*n*	*islands*	*gens*	*individuals*	*migration*	*pcross*	*pmut*	*p*
4	24	250	200	10	[0.5, 0.8]	[0.001, 0.002]	[0.6, 0.9]

We have used this EA to find 14 efficient binary classifiers—one for each spoligotype family—to predict based on binary masks combination. Since our approach is a binary classification method by nature, we have followed a ‘OVA’ strategy to integrate them into a single multiclass classifier. Each of these classifiers takes as input specific relabelled datasets where only one family is kept and all the other families are changed to ‘Other’. When a new example has to be classified, it is given successively as input to each binary classifier. The final answer corresponds to that of the classifier that detects a family. A priority table obtained through an optimization technique based on the answers of each classifier on the learning dataset is used to make a single prediction when two or more binary classifiers detect different families.

### Comparative performance of models

Then we have compared the different existing approaches (Expert rules, TB-Lineage and Borile AP) to the two approaches we propose DT (with 8 and 43 attributes, respectively, denoted *DT 8* and *DT 43*) and binary masks with an EA (denoted *EA*). Table 4 presents the overall model performances for ‘true positive’ (TP) and ‘false positive’ (FP) rates and ‘precision’. For each indicator, the values of the three best models are shown in bold.

**Table 4.** Overall model performance on the testing set

	Expert rules	TB- Lineage	Borile AP	DT 8	DT 43	EA
TP rate	0.993	0.937	0.835	0.978	0.982	0.951
FP rate	0.011	0.020	0.108	0.004	0.002	0.028
Precision	0.989	0.979	0.886	0.979	0.983	0.970

Regarding the ‘TP rate’, we can globally observe that all models provide a high value, namely >90%, with the exception of Borile AP, which has a TP rate of about 85%. Three best results are obtained for Expert rules, DT 8 and DT 843. Trends are the same for ‘FP rate’ and model ‘precision’.

Thus, these results demonstrate the good performance of DTs in the task of classifying MTBC families. In particular, even when the spacers are reduced to eight attributes the model retains very good performance, with a precision of about 98%. Finally, if the EA is not in the top three, we can still note the good performance of the binary masks approach with a precision of about 97%.

To further understand these results, we have also compared the performance of models in the classification of each MTBC spoligotype family. Table 5 details the ‘precision’ of models for 14 families in the dataset.

**Table 5.** The precision of models on MTBC spoligotype families. For each family, the models with the best precision are displayed in bold

	Expert rules	TB-lineage	Borile AP	DT 8	DT 43	EA
Beijing	**1**	**1**	**1**	**1**	**1**	0.645
Bovis	**1**	0.92	0.808	0.966	0.978	0.997
Cameroon	**1**	**1**	0.846	**1**	0.963	**1**
CAS	**1**	0.961	0.967	0.983	0.994	0.972
EAI	0.987	0.969	0.964	**0.996**	0.993	0.973
H	**1**	**1**	0.871	0.986	0.994	0.983
LAM	0.951	**0.993**	0.829	0.991	0.986	0.939
Manu	0.994	-	-	**1**	0.993	**1**
PINI	**0.778**	-	0.333	0.353	0.267	0.666
S	0.985	**1**	0.667	0.984	0.908	**1**
T	**0.999**	0.996	0.954	0.973	0.990	0.982
Turkey	**1**	**1**	-	0.933	0.933	0.666
Ural	**1**	**1**	0.52	0.971	**1**	0.970
X	**0.991**	0.974	0.889	0.966	0.943	0.973

Comprehensively, good results of the models can also be seen in the classification of different families. Indeed, except the *PINI* family for which the results are rather bad, the methods compared are largely concordant since they present precision rates varying from 70% to 90% for the other MTBC spoligotype families. It demonstrates the effectiveness of all of these approaches in the classification task.

When comparing performances between different approaches, some disparities can be observed. For instance, less concordant results are observed for prediction made by Borile AP. This difference can be explained by the fact that some lineages, like Turkey and NEW-1 (previously misnamed as Ural-2), are not taken into account.

### Runtime evolution

**Figure 5.**
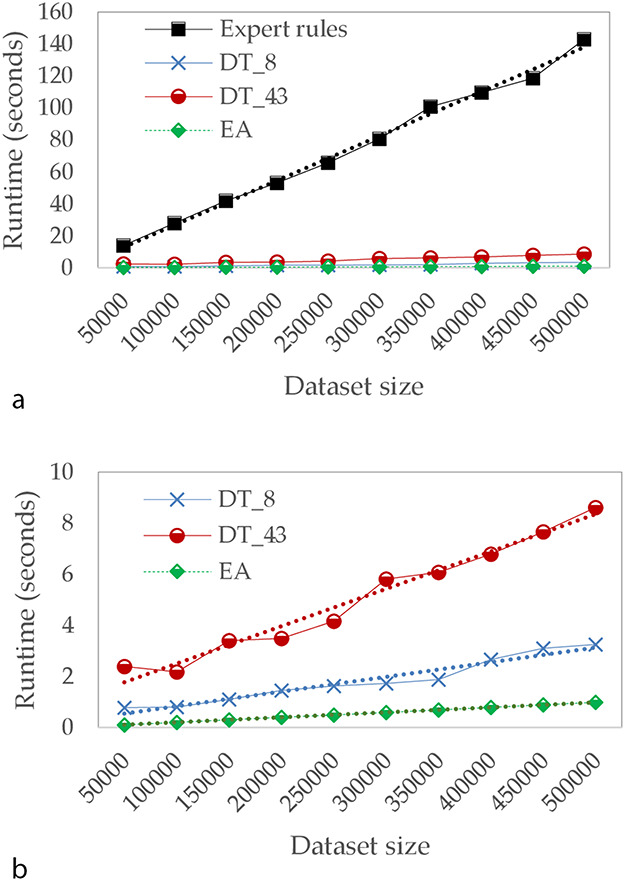
Comparison of runtime in seconds (a) for Expert rules and our approaches and (b) focused only on our approaches.

In the third part of our study, we focused on calculation times. Figure 5 describes the evolution of runtimes in seconds for different sizes of test datasets. For these tests, we assume that the model has already been learned and we only take into account the time needed to apply the model on test dataset. Runtime tests have been conducted on the following environment: Intel Core i7, 2.4 GHz, 32Go Ram, Linux Ubuntu 19.10.

Because of the input file size limit of TB-Lineage and TBminer (Borile AP) online tools, the comparison of calculation times has not been performed for these two approaches, since a lot of aspects (such as internet connection quality, performance of their web server, etc.) could have biased the results. As a result, we compare runtimes for Expert rules and the two approaches we propose—DT (with 8 and 43 attributes) and EA.

The computation time of all compared approaches increases linearly with the size of the dataset. However, when the dataset is large, the time required by Expert rules is significantly higher compared to other approaches. For instance, with 5 00 000 lines, Expert rules performs the classification in >120 seconds while <1 second is required for EA. We can also observe the impact of reducing the number of attributes on DTs.

These results on the computation time allow putting in perspective the performances observed in Table 4. Indeed, if the binary masks approach does not offer the best performances, it allows us to perform the classification very quickly. Consequently, binary masks based approach is undoubtedly the best compromise between speed of runtime and performance, since it still has an overall precision of 97%.

### Towards interpretable models

**Figure 6.**
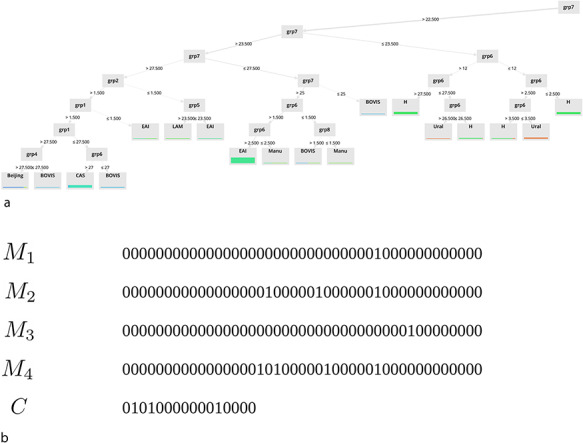
Examples of extracted models for (a) DT and (b) binary masks.

Finally, in the last part of our study, we focus on the interpretability of the models. Some examples of extracted models for (a) DT and (b) binary masks are shown in Fig. 6.

Note that only a subset of the underlying rules of these models is displayed on this graph. As you can observe, one of the main advantages of the two approaches we propose is their ability to provide simple classification rules that can be easily interpreted. These rules are available on the ‘Help’ page of the website.

## SpolLineages tool

To facilitate user analysis, we have decided to distribute SpolLineages as a freely available user-friendly command-line tool (https://github.com/dcouvin/SpolLineages) and as a web resource (http://www.pasteur-guadeloupe.fr:8081/SpolLineages). SpolLineages scripts are mainly written in Java and C programming languages. The online programme (using JavaServer Pages, HTML and JavaScript) allows users with no or few computing skills to easily perform their analysis based on spoligotyping and/or 24-loci MIRU-VNTR data. Users can either upload their CSV/TSV (separated by commas, tabs or semicolons) analysis file containing spoligotyping and/or 24-loci MIRU-VNTR data or directly enter their data in the provided text area. The output result file contains predictions of MTBC spoligotype families using our methods. It is also possible to get lineage prediction from 24-loci MIRU-VNTR data using rules provided in RuleTB (18). Other options allow users to get supplemental information such as Spoligotype International Type, country distribution (according to SITVIT2) and potential correspondence to SNP-based lineage [according to related research (5)]. Further details are provided on the web resource.

## Conclusion and future directions

To conclude, we propose novel algorithmic approaches allowing quick and precise prediction of MTBC genotypic families from spoligotyping data, using a DT, an EA or classical binary rules. These approaches are helpful for a better understanding and analysis of genotypic variability and evolution of MTBC. SpolLineages software tool (available online or via the command line) includes these methods and allows users to easily search for MTBC genotypic families provided their corresponding spoligotyping data are given. Indeed, this tool provides interpretable rules and a user-friendly interface, which could be helpful for the scientific community. In addition, data reduction methodologies were used to speed up calculations. Furthermore, the approaches presented here are of great importance for database improvement and development. Future works will consist of applying these algorithms (as well as novel ones) to predict better MTBC lineages from 24-loci MIRU-VNTR data (since for the moment, RuleTB intermediate rules are used for this task). Future development of additional software tools dealing with WGS data could be particularly helpful towards a better understanding of TB epidemiological diversity. Supplemental work will also be done to facilitate prediction of newly described MTBC lineages such as L8 and L9.
